# Patient with Unicuspid Aortic Valve and Ascending Aorta Aneurysm Treated with Ozaki Procedure and Ascending Aorta Replacement

**DOI:** 10.21470/1678-9741-2020-0150

**Published:** 2021

**Authors:** Antonios Roussakis, Simone Calvi, Eliana Raviola, Alberto Albertini

**Affiliations:** 1 Cardiothoracic and Vascular Department, Maria Cecilia Hospital, GVM Care & Research, Cotignola, (RA), Italy.; 2 1st Cardiac Surgery Department, Onassis Cardiac Surgery Center, Onassis Foundation, Athens, Greece.

**Keywords:** Aortic Valve Reconstruction, Unicuspid Valve, Ascending Aorta Aneurysm

## Abstract

Although aortic valve replacement remains the gold standard treatment for aortic valve diseases like stenosis (AS) or insufficiency, new surgical methods have been developed with a focus in the reconstruction of the aortic valve rather than replacing it. The Ozaki procedure involves a tailored replacement of each individual valvular leaflet with glutaraldehyde-treated autologous pericardium and aims to reproduce the normal anatomy of the aortic valve. Cases of patients with unicuspid aortic valve treated with the Ozaki procedure are uncommon in the litrature and become even more rare when it comes to concomitant diseases like AS and ascending aorta aneurysm.

We present the case of a 21-year-old, fit and asymptomatic male, with unicuspid aortic valve with severe stenosis and ascending aorta dilatation, surgically treated with tricuspidization of the aortic valve with glutaraldehyde-treated autologous pericardium and replacement of the ascending aorta with a straight synthetic graft. Postoperative studies showed a fully functional, neo-tailored tricuspid aortic valve with trivial regurgitation. The patient had an uncomplicated recovery, stayed in the intensive care unit for 2 days and was discharged on the 7th postoperative day.

**Table:** 

Abbreviations, acronyms & symbols	
AI	= Aortic valve insufficiency
AS	= Aortic valve stenosis
AVR	= Aortic valve replacement
CPB	= Cardiopulmonary bypass
CT	= Computed tomography
ICU	= Intensive care unit

## Case Presentation

Aortic valve disease is the most common acquired heart valve disease in Western countries, with aortic valve stenosis (AS) being more common when compared to aortic valve insufficiency (AI). For more than 30 years, open surgical aortic valve replacement (AVR) has been the gold standard treatment for symptomatic severe AS^[1,2]^. Mechanical valves are the preferred choice for young patients due to their durability over time, whereas bio-prostheses are used for elderly patients with the benefit of avoiding oral anticoagulation^[2]^. New, less invasive techniques like transcatheter aortic valve implantation have emerged over the last years, with the main target group of high-risk patients for conventional surgical AVR. Even more recently, new surgical methods have been developed with a focus on reconstructing the aortic valve rather than replacing it. Ozaki and colleagues published their technique in 2011, describing a tailored replacement of each individual valvular leaflet with glutaraldehyde-treated autologous pericardium, aiming at reproducing the normal anatomy of the aortic valve^[3]^.

Cases of patients with unicuspid aortic valve treated with the Ozaki procedure are uncommon in the literature and become even more rare when it comes to concomitant diseases like AS and ascending aorta aneurysm^[4,5]^.

**Fig. 1:**
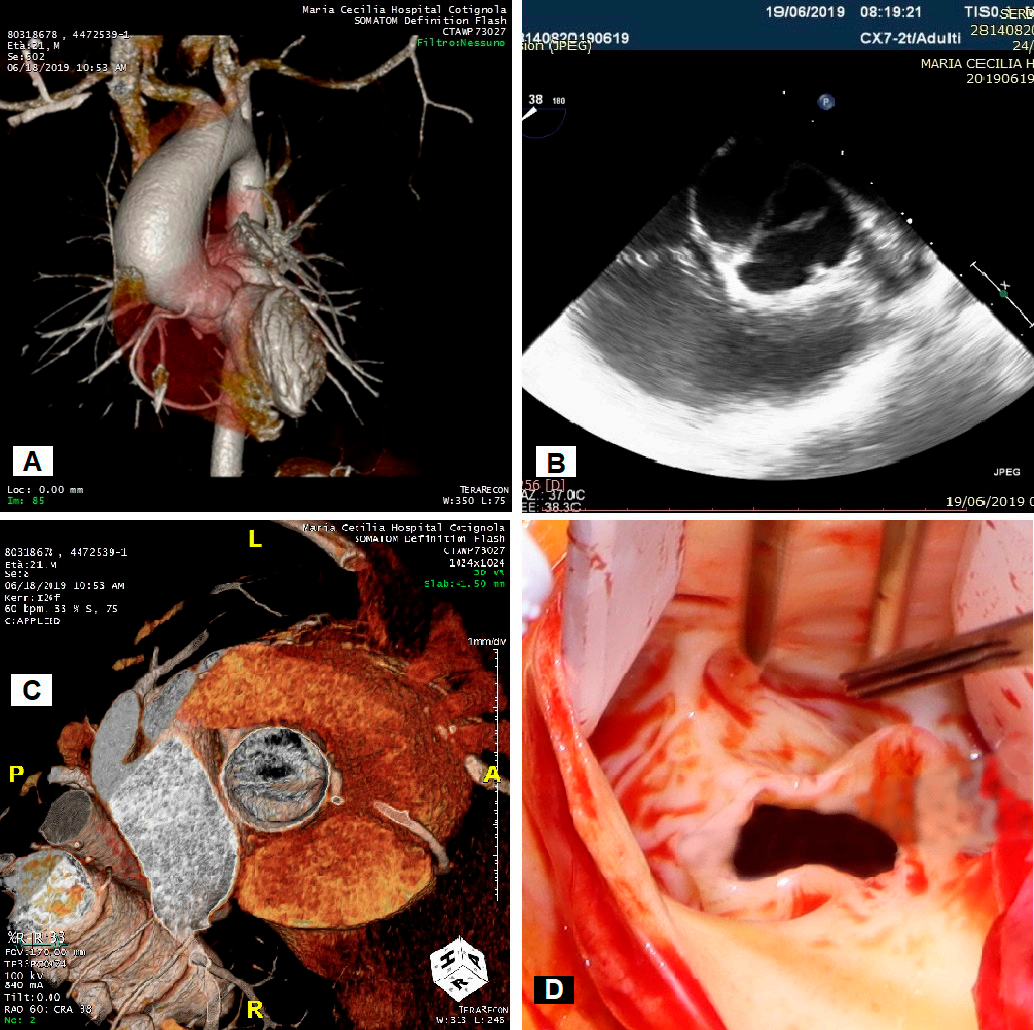
Pre- and intra-operative imaging. (A) Chest CT scan with 3D reconstruction - General view of the heart and aorta before repair. (B) Preoperative trans-oesophageal echocardiogram. (C) Chest CT scan with 3D reconstruction - Planar view of the unicuspid aortic valve. (D) Surgical view of the unicuspid aortic valve.

We present a case of a 21-year-old, fit and asymptomatic male, with a known unicuspid aortic valve with moderate stenosis and ascending aorta dilatation under surveillance. His last regular annual follow-up echocardiogram revealed progress of aortic valve stenosis to a level that was becoming severe (mean gradient 52 mmHg). Findings were confirmed with a preoperative trans-oesophageal echocardiogram and a chest computed tomography scan with 3D reconstruction showed the ascending aorta at the level of the pulmonary artery trunk measuring 52 × 51 mm (Figure 1A, 1B and 1C) (Video 1). Due to his young age, the patient was surgically treated with tricuspidization of the aortic valve with glutaraldehyde-treated autologous pericardium (Ozaki procedure) and replacement of the ascending aorta with a straight synthetic graft (IRB approval: 04/20-11-2012).

**Video 1:**
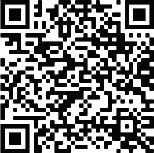
Preoperative transthoracic echocardiogram.

## Technical Description

The approach was performed through a median full sternotomy followed by autologous pericardium harvesting. A 70 × 90 mm piece of pericardium was harvested and treated with 0.6% glutaraldehyde solution according to the procedure protocol.

Standard cannulation of the distal ascending aorta and the right atrium was performed to establish cardio-pulmonary bypass (CPB). Custodiol cardioplegia was used for cardiac protection. Post-aortotomy intraoperative findings were constant in the preoperative imaging (Figures 1D and 3).

**Figure:**
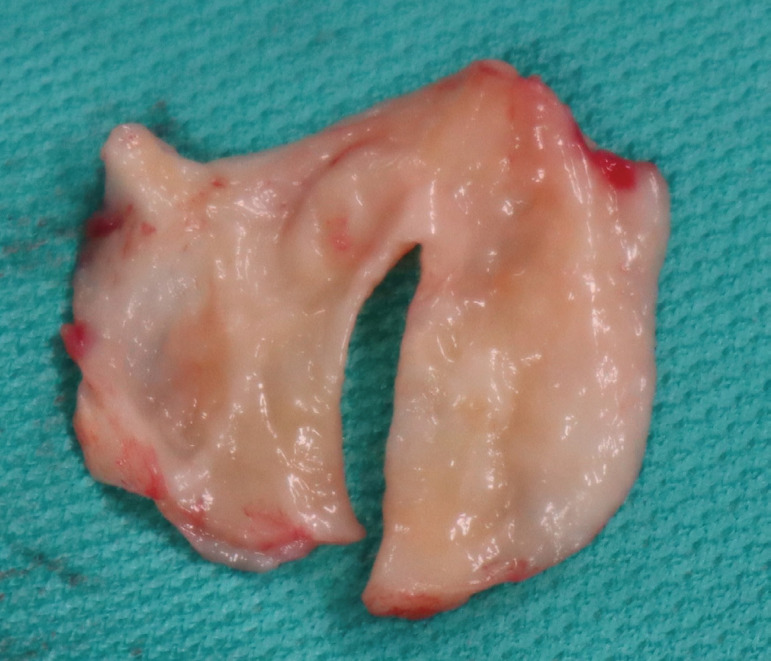

**Fig. 2:**
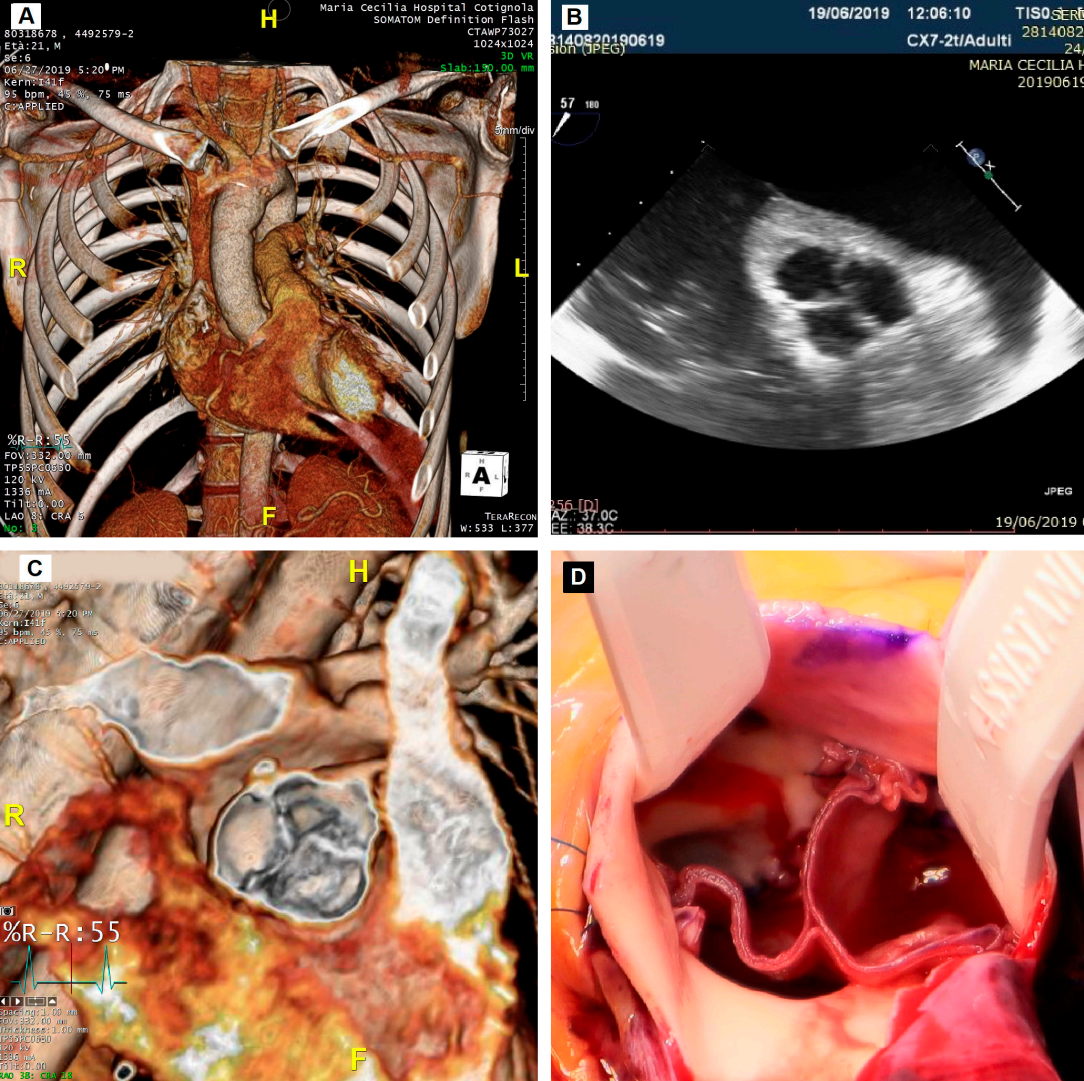
Post- and intra-operative imaging. (A) Chest CT scan with 3D reconstruction - General view of the heart and the aorta after repair. (B) Postoperative trans-oesophageal echocardiogram. (C) Chest CT scan with 3D reconstruction - Planar view of the neo-tailored tricuspid aortic valve. (D) Surgical view of the neo-tailored tricuspid aortic valve.

The diseased aortic valve was carefully excised. The annulus was sized with the original sizer from the OZAKI AVNeo Sizer System and revealed a 27-mm size for each of the remodeled cusps. Glutaraldehyde-treated autologous pericardium was trimmed to produce 3 identical cusps by using the original template. The new pericardial cusps were sutured in the aortic annulus using 4-0 Prolene sutures with a 13 mm HR needle, following Dr. Ozaki's technique. The surgical result was declared very satisfactory (Figure 2D).

After dissection and sizing of the ascending aorta, a standard aortic replacement technique with a straight Vascutek^®^ Gelsoft™ 24 mm synthetic graft took place.

The CPB time was 154 minutes and the aortic cross-clamp time was 141 minutes.

Intraoperative trans-oesophageal and postoperative trans-thoracic echocardiograms and the postoperative chest computed tomography scan with 3D reconstruction showed a fully functional neo-tailored tricuspid aortic valve with trivial regurgitation (Figure 2A, 2B and 2C) (Video 2) (small jet originating from the commissure between the left and non-coronary leaflet), mean gradient 7 mmHg and excellent coaptation zone. The patient had an uncomplicated recovery, stayed in intensive care unit for 2 days and was discharged on the 7^th^ postoperative day. In the 6-month follow-up appointment, the patient was free from any symptoms, able to manage his daily routine without any problem and the transthoracic echo showed maintenance of the excellent findings and elimination of the small residual regurgitant jet noted in the immediate postoperative period.

**Video 2:**
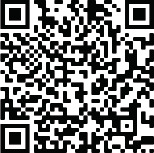
Postoperative transthoracic echocardiogram.

## Comments

The main advantages of the Ozaki procedure include avoidance of oral anticoagulation, avoidance of foreign protheses, suitability for young patients and suitability for patients with a small aortic annulus. The mid-term results of reconstructed aortic valve in 850 patients were published in 2018 and included all types of diseases (AS, AI, mixed diseases) and all types of anatomies (tricuspid, bicuspid, unicuspid and quadricuspid aortic valves). The mean follow-up period in this study was 53.7±28.2 months, and the longest follow-up was 118 months. This study confirmed the hemodynamic advantages of the method with very low transvalvular gradient and a large effective orifice area of the new valve. It also presented 85.9% freedom of death, 4.2% cumulative incidence of reoperation, and 7.3% recurrent moderate or greater aortic regurgitation during this follow-up period^[4]^. The durability of the neo-tailored valve, although it seems very promising from the mid-term follow-up, remains to be proven in the long term.

In conclusion, we would like to state that the Ozaki procedure can be an excellent alternative for aortic valve surgery even for rare (e.g. unicuspid valve) or concomitant (e.g. ascending aorta aneurysm) diseases. The data available up to now shows excellent results for this type of procedure, but further evidence from long-term studies and randomized clinical trials are required to reach a safer conclusion regarding the place of the Ozaki procedure among the other surgical strategies for the aortic valve.

**Table:** 

Authors' roles & responsibilities	
AR	Substantial contributions to the conception or design of the work; or the acquisition, analysis or interpretation of data for the work; drafting the work or revising it critically for important intellectual content; final approval of the version to be published
SC	Drafting the work or revising it critically for important intellectual content; final approval of the version to be published
ER	Substantial contributions to the conception or design of the work; or the acquisition, analysis or interpretation of data for the work; final approval of the version to be published
AA	Substantial contributions to the conception or design of the work; or the acquisition, analysis or interpretation of data for the work; drafting the work or revising it critically for important intellectual content; final approval of the version to be published

## References

[1] Nkomo VT, Gardin JM, Skelton TN, Gottdiener JS, Scott CG, Enriquez-Sarano M (2006). Burden of valvular heart diseases: a population-based study. Lancet.

[2] Vahanian A, Alfieri O, Andreotti F, Antunes MJ, Barón-Esquivias G, Baumgartner H (2012). Guidelines on the management of valvular heart disease (version 2012): the joint task force on the management of valvular heart disease of the European society of cardiology (ESC) and the European association for cardio-thoracic surgery (EACTS). Eur J Cardiothorac Surg.

[3] Ozaki S, Kawase I, Yamashita H, Uchida S, Nozawa Y, Matsuyama T (2011). Aortic valve reconstruction using self-developed aortic valve plasty system in aortic valve disease. Interact Cardiovasc Thorac Surg.

[4] Ozaki S, Kawase I, Yamashita H, Uchida S, Takatoh M, Kiyohara N (2018). Midterm outcomes after aortic valve neocuspidization with glutaraldehyde-treated autologous pericardium. J Thorac Cardiovasc Surg.

[5] Tokue M, Hara H, Sahara N, Yamazaki K, Yamashita H, Takahashi K (2015). A case of severe unicuspid aortic valve stenosis: valve repair with tricuspidization in an adult. World J Pediatr Congenit Heart Surg.

